# Sickle cell cerebrovascular reactivity to a CO_2_ stimulus: Too little, too slow

**DOI:** 10.3389/fphys.2022.886807

**Published:** 2022-08-19

**Authors:** Stéphanie Forté, Olivia Sobczyk, Julien Poublanc, James Duffin, Gregory M. T. Hare, Joseph Arnold Fisher, David Mikulis, Kevin H. M. Kuo

**Affiliations:** ^1^ Division of Medical Oncology and Hematology, Departement of Medicine, Centre Hospitalier de l'Université de Montréal, Montréal, QC, Canada; ^2^ Division of Medical Oncology and Hematology, Department of Medicine, University Health Network, Toronto, ON, Canada; ^3^ Division of Hematology, Department of Medicine, University of Toronto, Toronto, ON, Canada; ^4^ Joint Department of Medical Imaging, University Health Network, Toronto, ON, Canada; ^5^ Department of Anaesthesia and Pain Medicine, University Health Network, Toronto, ON, Canada; ^6^ The Keenan Research Centre for Biomedical Science, Li Ka Shing Knowledge Institute, St. Michael’s Hospital, Toronto, ON, Canada; ^7^ Department of Physiology, University of Toronto, Toronto, ON, Canada; ^8^ Department of Anesthesia, St. Michael’s Hospital, Toronto, ON, Canada; ^9^ Institute of Health Policy, Management and Evaluation, Dalla Lana School of Public Health, University of Toronto, Toronto, ON, Canada

**Keywords:** sickle cell disease (SCD), cerebrovascular reactivity (CVR), cerebrovascular reactivity to carbon dioxide, functional magnetic resonance imaging (fMRI), cerebral hemodynamics, cerebral blood flow (CBF), cerebrovascular disorders

## Abstract

**Background:** Despite increased cerebral blood flow (CBF), cerebral infarcts occur in patients with sickle cell disease (SCD). This suggests increased CBF does not meet metabolic demand possibly due to compromised cerebral vasodilatory response. Hypothesis: In adult SCD patients, cerebrovascular reactivity (CVR) and speed of vasodilatory response (tau) to a standardized vasodilatory stimulus, are reduced compared to normal subjects.

**Methods:** Functional brain imaging performed as part of routine care in adult SCD patients without known large vessel cerebral vasculopathy was reviewed retrospectively. CVR was calculated as the change in CBF measured as the blood-oxygenation-level-dependent (BOLD)-magnetic resonance imaging signal, in response to a standard vasoactive stimulus of carbon dioxide (CO_2_). The tau corresponding to the best fit between the convolved end-tidal partial pressures of CO_2_ and BOLD signal was defined as the speed of vascular response. CVR and tau were normalized using a previously generated atlas of 42 healthy controls.

**Results:** Fifteen patients were included. CVR was reduced in grey and white matter (mean Z-score for CVR −0.5 [−1.8 to 0.3] and −0.6 [−2.3 to 0.7], respectively). Tau Z-scores were lengthened in grey and white matter (+0.9 [−0.5 to 3.3] and +0.8 [−0.7 to 2.7], respectively). Hematocrit was the only significant independent predictor of CVR on multivariable regression.

**Conclusion:** Both measures of cerebrovascular health (CVR and tau) in SCD patients were attenuated compared to normal controls. These findings show that CVR represents a promising tool to assess disease state, stroke risk, and therapeutic efficacy of treatments in SCD and merits further investigation.

## 1 Introduction

Sickle cell disease (SCD) compromises cerebrovascular vascular health. By age 30, over 50% of SCD patients have suffered a cerebral infarct (Kassim et al., 2016). In response to anemia and the reduction in oxygen-carrying capacity, cerebral blood flow (CBF) increases to meet the metabolic demand. Increased velocity of CBF in major cerebral arteries is a strong risk factor for stroke in SCD children and adolescents (Adams et al., 1998). Despite generally increased CBF, silent cerebral infarcts (SCI) can still occur in patients receiving optimal transfusions (Hulbert et al., 2011). SCI occur mostly in border zones, where blood supply is precarious (Ford et al., 2018). Overall, this suggests that the increased CBF does not meet the metabolic demand and may be compromising cerebrovascular health. In support of this hypothesis, treatment with red blood cell transfusion has been shown to restore cerebrovascular reactivity (CVR) in children (Kosinski et al., 2017) and reduce the incidence of stroke and SCI in patients with SCD (Adams et al., 1998; DeBaun et al., 2014).

Treatment of SCD focuses on prevention through regular blood transfusions that decrease stroke risk (DeBaun et al., 2020). However, transfusion therapy is only initiated after a stroke has occurred or if patients are deemed high risk as determined by elevated transcranial Doppler velocity or SCI in childhood. Waiting for these late signs of injury risk, may result in missed opportunities for preventive therapeutic intervention. Additional research is needed to focus on the brain vascular health to determine if identification of early cerebrovascular dysfunction, and early treatment intervention, can further reduce the incidence of SCI and stroke in adult patients.

Brain vascular health may be assessed indirectly using measurements of CVR (Mandell et al., 2008). CVR is measured by analyzing the CBF response to a vasodilatory stimulus such as an increase in arterial partial pressure of carbon dioxide (PaCO_2_). CVR has been used to examine the cerebrovascular health in patients with steno-occlusive disease (Mandell et al., 2008), cognitive deficits (Balucani et al., 2012) and concussion (Shafi et al., 2021). Impaired CVR has been shown to be associated with the progression of white matter hyperintensities in aging adults (Sam et al., 2016). Slowed temporal and parietal responses were also described in amnestic mild cognitive impairment add early Alzheimer’s disease (Holmes et al., 2020). Using a similar approach, Leung et al. showed an altered temporal dynamic of vascular response in children with SCD (Leung et al., 2016). However, this has not yet been applied to quantify and map cerebrovascular health in adults with SCD without known large vessel steno-occlusive cerebrovascular disease.

We hypothesized that in adult patients with SCD, CVR and the speed of the vasodilatory response (tau) (Poublanc et al., 2015) to a standardized vasodilatory stimulus (CO_2_), are reduced compared to healthy subjects. Our aim was to explore possible associations of CVR and tau with clinical and biological characteristics.

## 2 Methods

### 2.1 Ethics approval and reporting guidelines

This study conformed to the standards set by the latest revision of the Declaration of Helsinki and were approved by the Research Ethics Board of the University Health Network. Patients were participants in prospective studies in which CVR was measured. All patients provided written and informed consent for the original studies.

For the manuscript preparation, we followed the Strengthening the Reporting of Observational Studies in Epidemiology (STROBE) reporting guideline.

### 2.2 Study design

We conducted a single center, retrospective, cross-sectional study.

### 2.3 Setting

Participants were drawn for the SCD comprehensive care center at the University Health Network (Toronto, Canada).

### 2.4 Participants

Adult patients (≥18 years), with any SCD genotype were included if they had undergone functional brain imaging performed as part of routine care between 1 January 2017 and 20 November 2018. They were excluded if they had known cerebrovascular disease (e.g., large vessel stenosis, Moya Moya).

### 2.5 Data sources, variables and measurements

Patient characteristics, biological variables and radiological findings were extracted from the electronic patient record.

### 2.6 Clinical variables

Sickle cell disease comorbidities and complications were defined as previously reported (Sebastiani et al., 2007). The SCD severity score was computed using the online calculator created by Sebastiani et al. (Sebastiani et al., 2007) (Sickle cell disease severity calculator. Accessed 21 May2021. http://bios.ugr.es/dss-calculator/). Systolic blood pressure was recorded immediately prior to functional brain imaging.

### 2.7 Laboratory markers

For laboratory variables, most recent values, within 1 year of the functional brain imaging, of hemoglobin, hematocrit, mean corpuscular volume, lactate dehydrogenase, reticulocytes, white blood cells (WBC), neutrophiles, monocytes, lymphocytes, platelets, bilirubin, fetal hemoglobin fraction (HbF), globular filtration rate (GFR) and urine protein were recorded. GFR was approximated using the Modification of Diet in Renal Disease Study equation.

### 2.8 Definitions of silent cerebral infarct and white matter hyperintensities on MRI

SCI were defined as lacunae at least 3 mm in one dimension, visualized on at least 2 planes on Fluid-attenuated inversion recovery (FLAIR) (DeBaun et al., 2014). White matter hyperintensities were defined as abnormal signals on T2 FLAIR.

### 2.9 CVR Experimental protocol

#### 2.9.1 Hypercapnic stimulus

**FIGURE 1:**
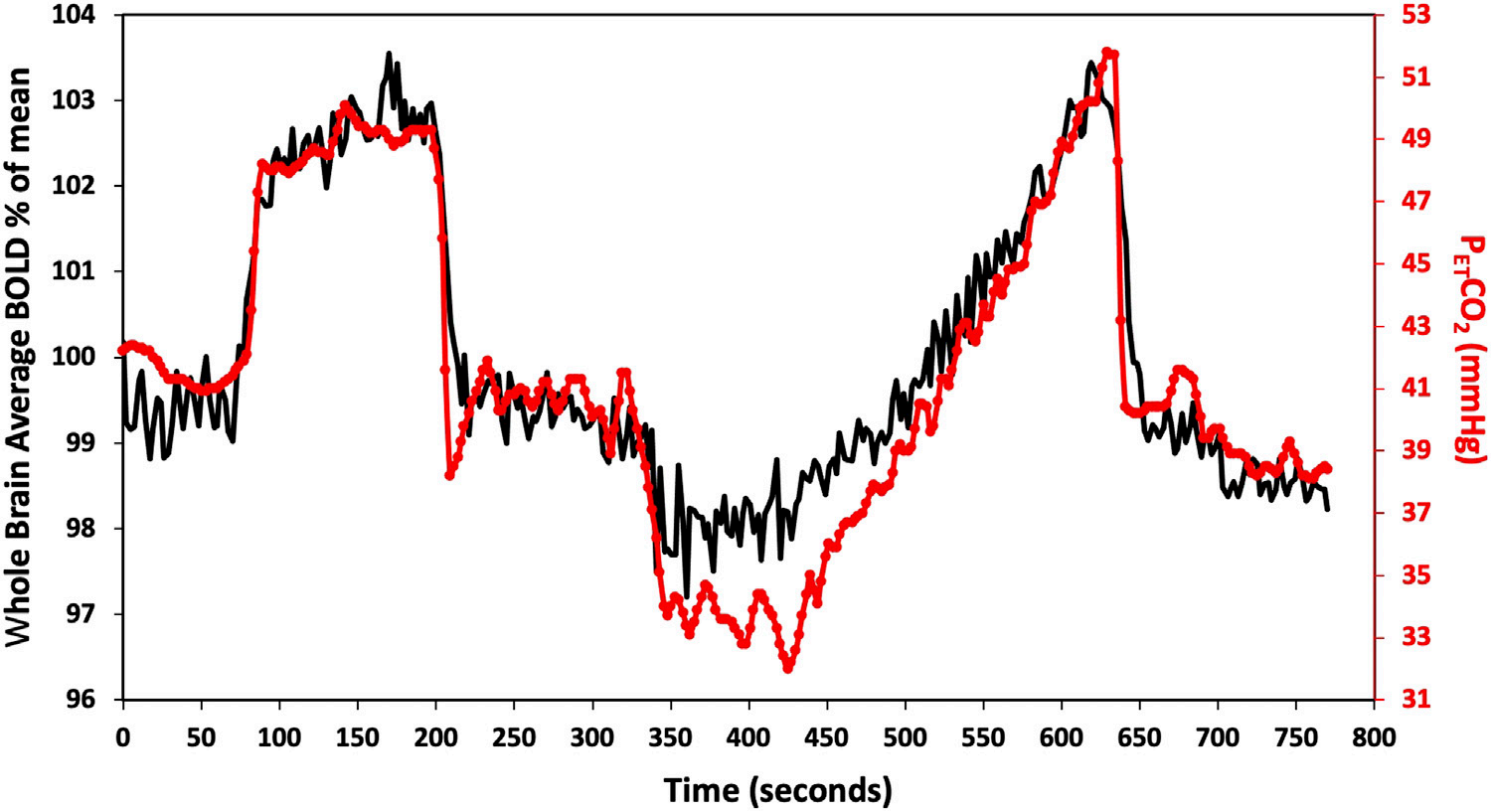
Standard P_ET_CO_2_ sequence in a representative subject. The CO_2_ stimulus (red line), and whole brain average BOLD response (black line) are shown. CVR metrics (tau and CVR) were calculated for the step portion of the response.

Participants underwent a standardized hypercapnic isoxic stimulus using a computer-controlled gas blender (RespirAct, Thornhill Research Inc., Toronto, Canada) running a prospective gas targeting algorithm (Slessarev et al., 2007), that controls CO_2_ where end-tidal partial pressures of CO_2_ (P_ET_CO_2_) is equivalent to arterial partial pressures of CO_2_ PaCO_2_ (Ito et al., 2008). The stimulus consisted of clamping P_ET_CO_2_ at the patients resting level for 1 min, followed by a step increase by 10 mmHg for 2 min and returned to baseline for another 2 min. P_ET_CO_2_ was then reduced from baseline by 10 mmHg for 1 min, followed by a steady rise in P_ET_CO_2_ to 15 mmHg above baseline over ∼5 min, and returned to baseline for 2 min as shown in Figure 1 (Duffin et al., 2017).

#### 2.9.2 Imaging protocol and CVR post processing

During the vasodilatory stimulus, CBF changes were measured using blood-oxygen-level-dependent (BOLD) MRI. MR imaging was performed with a 3T scanner (Signa HDx; GE Healthcare, Milwaukee, Wisconsin) with an 8-channel phase-array head coil and consisted of a BOLD acquisition using a gradient echo pulse sequence with echoplanar readout (TR = 2,400 ms, TE = 30 ms, flip angle = 70°, 41 slices, voxel size = 3.5 mm^3^ isotropic, matrix size = 64 × 64, number of volumes = 335, field of view = 24 × 24 cm, acquisition time = 13.4 min). The acquired MRI and P_ET_CO_2_ data were analyzed using Analysis of Functional NeuroImaging software (AFNI, National Institutes of Health, Bethesda, Maryland; http://afni.nimh.nih.gov/afni) (Cox, 1996). BOLD images were first volume registered and slice-time corrected and coregistered to a high-resolution 3D T1-weighted Inversion-Recovery Prepared Fast Spoiled Gradient Echo (IR-FSPGR) volume (TI = 450 ms, TR = 7.88 ms, TE = 3 ms, flip angle = 12°, voxel size-0.859 × 0.859 × 1 mm, matrix size = 256 × 256, 146 slices, field of view = 24 × 24 cm, no interslice gap, acquisition time = 7.4 min) that was acquired during the same scan session. P_ET_CO_2_ data was aligned to the whole brain average BOLD signal and a linear, least-squares fit of the BOLD signal data series to the P_ET_CO_2_ data series was performed on a voxel-by-voxel basis, together with a linear trend regressor to calculate CVR values. This method is described in greater detail elsewhere (Fierstra et al., 2010; Sobczyk et al., 2014). CVR values were color-coded based on a spectrum of colors denoting the magnitude of response to create CVR maps. Additional CVR metrics were calculated. The dynamic (tau) component of the BOLD signal response was calculated based on the step portion of the stimulus. The P_ET_CO_2_ waveform was convolved with an exponential decay function. The exponential corresponding to the best fit between the convolved P_ET_CO_2_ and BOLD signal was defined as the speed of vascular response. This method is described in greater detail in Poublanc et al. (Poublanc et al., 2015).

**FIGURE 2:**
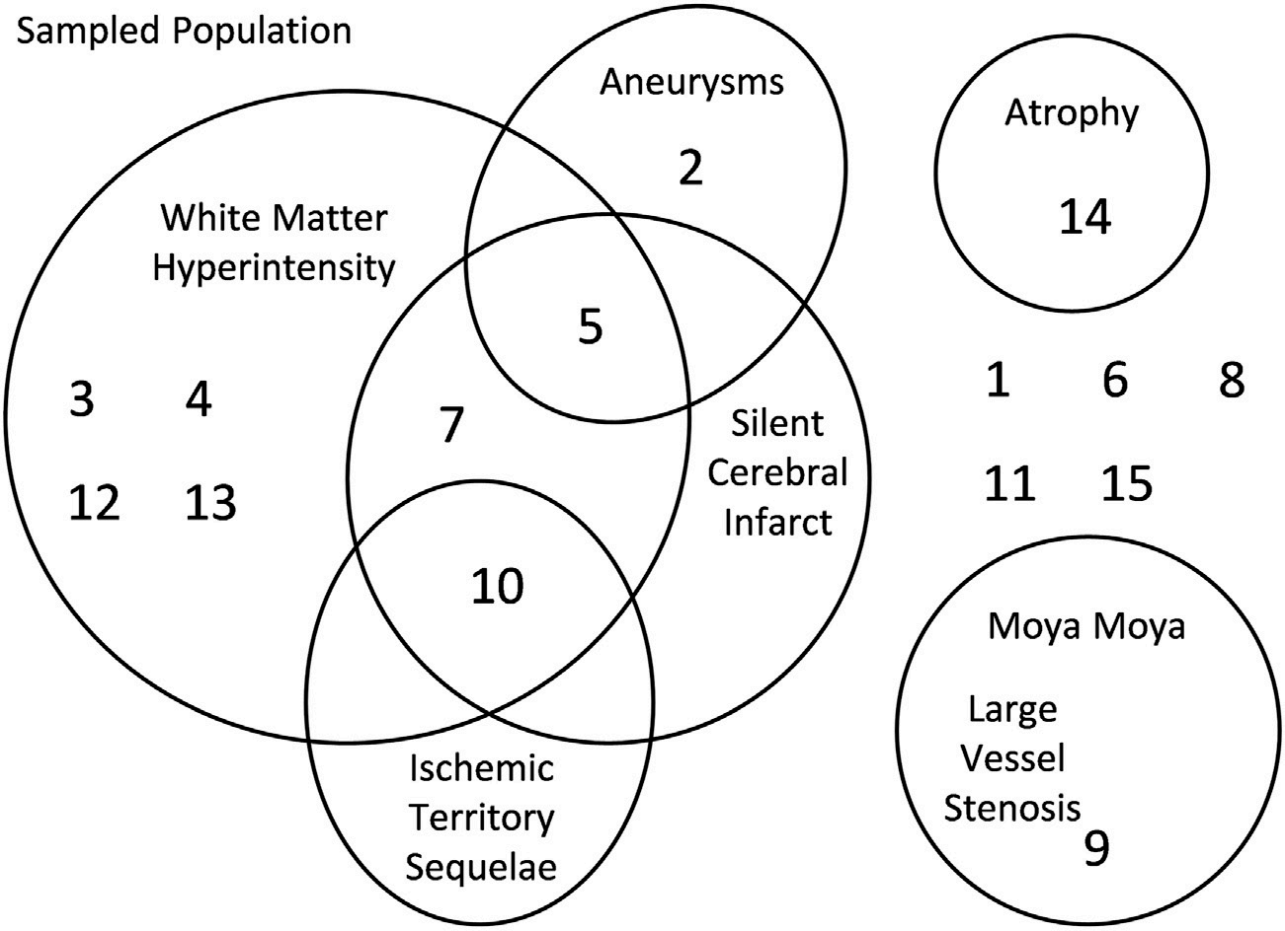
Anatomic findings on magnetic resonance imaging/angiography.

CVR and tau were normalized by calculating voxel-wise Z-scores relative to the mean and standard deviation of the same metric in the corresponding voxels of previously generated atlases of 42 age matched healthy controls that underwent the same CVR protocol (including the respiratory paradigm) as the SCD patients (McKetton et al., 2018; Holmes et al., 2020). The Z-values were then color coded and superimposed on the anatomical scans to form site-specific Z-maps, using a methodology described previously (Sobczyk et al., 2015). The co-registered T1-weighted anatomical images for all subjects were segmented into grey and white matter (GM and WM) using SMP12 software [SPM12: Wellcome Department of Imaging Neuroscience, University of London, United Kingdom; http://www.fil.ion.ucl.ac.uk/spm/software/spm12]. These masks were used to calculate mean GM and WM values for CVR and tau in each subject. For illustration purposes, only in the maps found in Figure 2, we provide a visual broad overview with a lower-resolution color scale to the reader*.* Here the color scheme bundles z scores broadly into no color for values −1< *x* < 1 (as we feel these values show no gross clinically significant changes from normal), values from 1 to 2; 2 to 3; >3 and −1 to −2; −2 to −3; −3 + to highlight gross changes.

### 2.10 Confounding and bias

To avoid confounding by acute medical events, such as vaso-occlusive crises, we excluded blood work that was drawn less than 3 weeks from an emergency department visit or inpatient admission.

Patients were scanned within 7 days prior to the scheduled transfusion so that the results were obtained at the participants’ baseline to minimize confounding.

### 2.11 Study size

For this proof of principle analysis, no sample size was calculated.

### 2.12 Statistical methods

For binary and categorical variables, t- and F-statistic were used to test for associations with CVR metrics. For continuous variables, Pearson r coefficients were calculated. For binary and categorical variables, effect sizes are reported with the Cohen d and η2 statistics, respectively. Univariate analysis was performed to identify possible associations between CVR metrics and 19 predetermined variables chosen for their ability to predict mortality in SCD patients based on a Bayesian network analysis (Sebastiani et al., 2007). These variables are age, sex, genotype, Sebastiani severity score, systolic blood pressure, organ complication score, hematocrit, hemoglobin, mean corpuscular volume, reticulocytes, lactate dehydrogenase, bilirubin, white blood cells, platelets, glomerular filtration rate, hemoglobin F fraction, anatomical findings on MRI/A, hydroxyurea and chronic transfusions. Associations with univariate *p* ≤ 0.2 were included in the multiple linear regression model. Multi-collinearity was assessed. No adjustment for multiplicity was made. Missing data was not imputed. Statistical analyses were conducted in SPSS (IBM, NY, United States).

## 3 Results

### 3.1 Participant’s characteristics

Of the 16 MRI/MRA + CVR, one was excluded due to poor imaging quality. We described the first 15 patients who underwent MRI/MRA + CVR. The median age was 27 [range 19–46] years; 5 (33.3%) were male. SCD genotypes were distributed as follows: 9 (60%) were SS or Sβ^0^ and 5 (33.3%) were SC. All patients were on disease modifying therapy: 11 (73.3%) were on hydroxyurea therapy and 4 (26.7%) were on transfusion. Only 1 (6.7%) had a prior history of stroke (Table 1). The control atlas was derived from 42 subjects with a mean age of 34.3 (13.6) years; 24 (57.1%) were male (Supplementary Table S1) (McKetton et al., 2018).

**TABLE 1:** Demographic, clinical, biological and radiologic characteristics of the 15 sickle cell disease patients with functional brain imaging between January 1, 2017 and November 20, 2018.

	Summary statistics
**Age (years)**
Median [IQR]	27 [22–35]
Range	19-46
**Sex (n (%))**
Male	5 (33.3)
Female	10 (66.7)
**Genotype (n (%))**
SS	9 (60.0)
SC	5 (33.3)
Sβ^+^	1 (6.7)
**Disease-modifying therapy (n (%))**
Hydroxyurea	11 (73.3)
Chronic exchange transfusions	4 (26.7)
**Clinical sickle cell complications (n (%))**
Acute chest syndrome	7 (46.7)
Priapism^a^	2 (40.0)
Sepsis	0
Stroke	1 (6.7)
Pain	15 (100.0)
Transfusion	9 (60.0)
Avascular necrosis	4 (26.7)
Sickle retinopathy	6 (40.0)
Restrictive lung disease	1 (6.7)
Pulmonary hypertension	1 (6.7)
Leg ulcers	0
Neurosensitive hearing loss	0
**Radiological findings on conventional MRI/MRA (n (%))**
Silent cerebral infarcts	3 (21.4)
White matter hyperintensities	7 (50.0)
Atrophy	1 (6.7)
Moya Moya	1 (6.7)
Aneurysms	2 (13.3)
No finding	5 (33.3)
**Laboratory parameters (mean ± SD, [range])**
Hemoglobin (g/L)	96 ± 16 [68–125]
Hematocrit (L/L)	0.27 ± 0.05 [0.19–0.35]
MCV (fL)	92 ± 14 [70–117]
LDH (U/L)	397 ± 198 [174–814]
Reticulocytes (%)	9.1 ± 6.4 [1.2–22.0]
WBC (10^9^/L)	7.4 ± 2.4 [3.0–11.8]
Platelets (10^9^/L)	308 ± 207 [72–875]
Bilirubin (mmol/L)	45.8 ± 37.1 [7.0–159.0]
HbF (%)	7.7 ± 6.2 [1.2–19.1]
Globular filtration rate (ml/min/1.73 m^2^)	113 ± 12 [84–120]
Urine protein (g/L)	0.11 ± 0.7 [0–0.28]
**Systolic blood pressure (mmHg) (mean ± SD, [range])**	120 (7) [103–131]
**Sebastiani severity score (%)**	14.4 ± 10.9 [3.2–49.2]

a Only males experience priapism.

Missingness: bilirubin 2 (13.3%), glomerular filtration rate 2 (13.3%), HbF 2 (13.3%), hematocrit 2 (13.3%), hemoglobin 1 (6.7%), LDH 6 (40.0%), MCV 2 (13.3%), platelets 2 (13.3%), reticulocytes 3 (20%), urine protein 6 (40.0%), WBC 1 (6.7%).

Abbreviations: HbF, hemoglobin F; IQR, interquartile range; LDH, lactate dehydrogenase; MCV, mean corpuscular volume; WBC, white blood cells.

There is considerable heterogeneity in the resting P_ET_CO_2_ in SCD, ranging from 33 to 47 mmHg (Supplementary Table S2). The P_ET_CO_2_ in SCD patients significant differed from controls (control vs. SCD patients, 37.8 (SD 3.9 vs. 40.9 (SD 4.1) respectively, *p* = 0.01). We therefore elected to use the patient’s own resting P_ET_CO_2_ rather than applying 40 mmHg uniformly across all patients.

### 3.2 Anatomical findings

**FIGURE 3:**
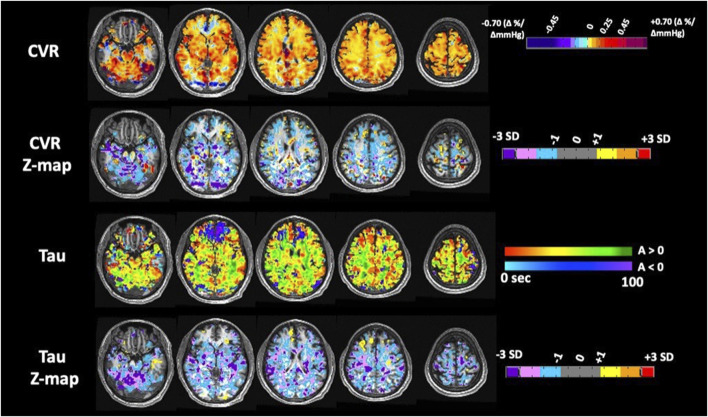
Axial images of CVR, amplitude (ssCVR) and tau measurements for subject 7with the corresponding z-maps created by scoring each parameter voxel-wise against a healthy control atlas.

MRI/MRA uncovered Moya moya in 1 patient. Ischemic lesions were distributed as follows: ischemic territory sequelae in 1 (6.7%) patient with a prior history of stroke, SCI in 3 (21.4%) and white matter hyperintensities in 7 (50.0%). Aneurysms were uncovered in 2 (13.3%) patients. Atrophy was described in 1 (6.7%) patient. Only 5 (33.3%) had none of these findings (Figure 3).

### 3.3 Primary outcomes

In Table 2, we present the summary CVR metrics for the grey matter and white matter. Compared to the reference atlas of normal subjects, CVR was reduced both in GM and WM (mean Z-score (SD) [range] −0.5 (0.6) [−1.8 to 0.3] and −0.6 (0.7) [−2.3 to 0.7], respectively). Tau was longer in GM and WM compared to normal subjects (mean Z-score (SD) [range] +0.9 (1.0) [−0.5 to 3.3] and +0.8 (0.9) [−0.7 to 2.7], respectively).

**TABLE 2:** Summary statistics of grey matter and white matter CVR.

	Grey matter	White matter
CVR, mean Z-score (SD) [range]	−0.5 (0.6) [−1.8 to 0.3]	−0.6 (0.7) [−2.3 to 0.7]
Tau, mean Z-score (SD) [range]	+0.9 (1.0) [−0.5 to 3.3]	+0.8 (0.9) [−0.7 to 2.7]

Abbreviations: CVR, cerebrovascular reactivity; SD, standard deviation.

### 3.4 Secondary outcomes

CVR metrics were mapped to the 6 vascular territories supplied by the left and right anterior, middle, and posterior cerebral arteries (Supplementary Table S3). Compared to the atlas of normal subjects, CVR was reduced and tau was longer across all 6 vascular territories.

### 3.5 Individual patient metrics

**FIGURE 4:**
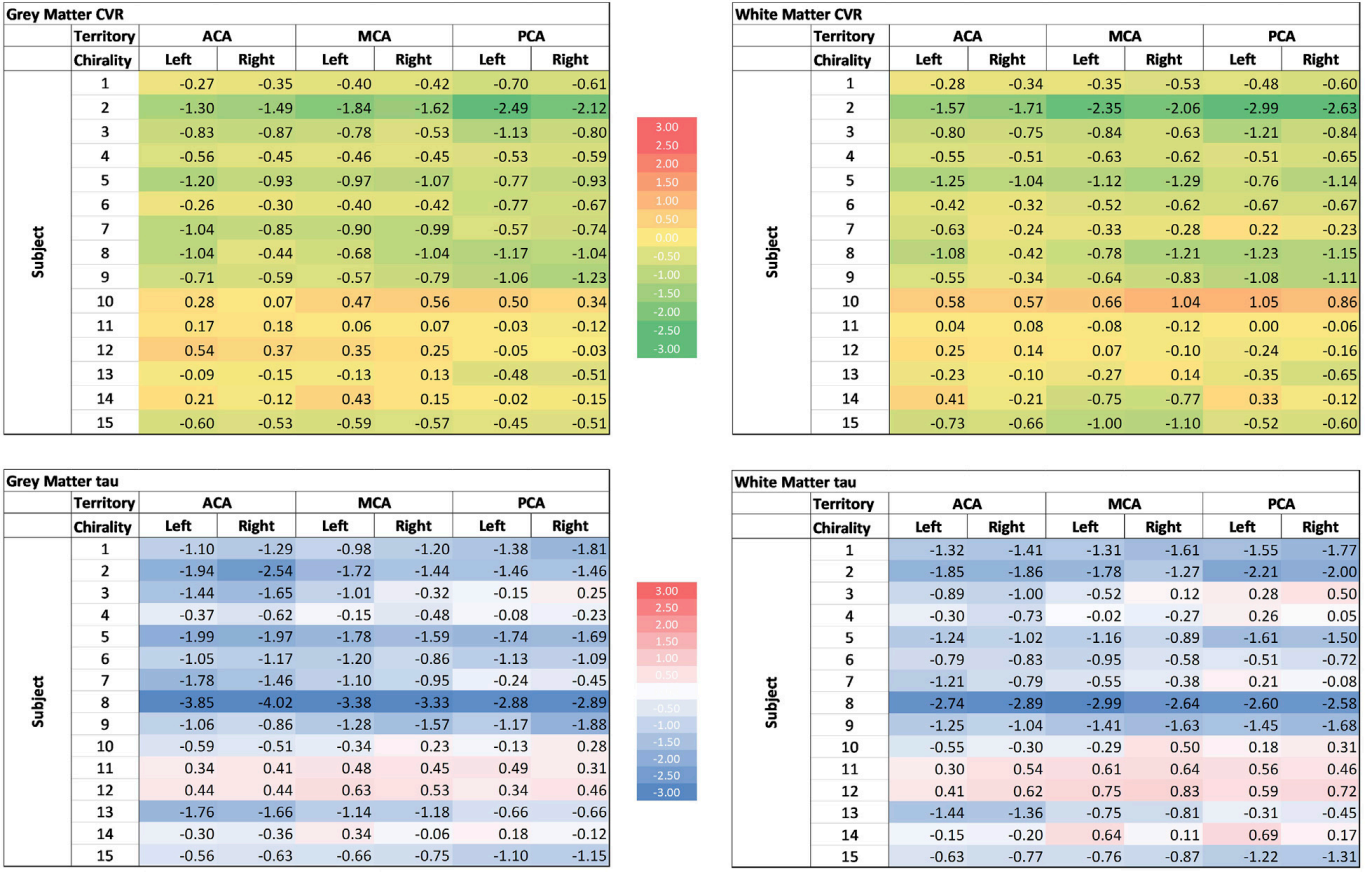
Cerebrovascular reactivity (CVR) and tau of different cerebral vascular territories in the grey and white matter. Colour scale is displayed in the middle. ACA: anterior cerebral artery; MCA: Middle cerebral artery; PCA: posterior cerebral artery.

We also computed individual patients’ regional Z-scores for CVR and tau. An example of a representative Z-map for patient 7 is shown (Figure 2). There was significant between-patient variability as illustrated by the wide ranges of Z-scores (Figure 4). Subjects 1 through 9 had a global reduction in CVR as well as reduction in all cerebral vascular territories in both the GM and WM. The degree of reduction between territories however was quite variable. The CVR in subjects 10 to 14 were more variable, with some territories showing a reduction in CVR while others showed an increased in CVR. In the gray matter, CVR is more heterogeneous across patients than tau (coefficient of variation −1.108 vs. −1.082 respectively); while in the white matter,; while in the white matter, tau is more heterogeneous across patients than CVR (coefficient of variation −1.201 vs. −1.052 respectively). While qualitatively it can be said that subjects with a reduction in CVR tend to have longer tau, there are considerable between-patient and within-patient variability. There was also no discernable trend in favour of a particular vascular territory across subjects.

### 3.6 Exploratory analyses

We explored associations between CVR and tau Z-scores and 19 predetermined variables, both for GM and WM (Tables 4-6). Only variables with *p*-values ≤0.2 on univariate analysis were selected to build multivariate analysis models.

#### 3.6.1 Univariate analysis

CVR and tau Z-scores were not significantly associated with the presence of an anatomical findings in the GM (t = −0.39, *p* = 0.70 and t = 0.91, *p* = 0.38, respectively) and WM (t = 0.06, *p* = 0.96 and t = 0.79, *p* = 0.44, respectively) (Table 3).

**TABLE 3:** Associations between mean grey and white matter CVR metrics and radiologic findings.

	CVR Z-score			Tau Z-score		
	Statistical test = statistic^a^	*p*-value	Effect size^b^	Statistical test = statistic^a^	*p*-value	Effect size^b^
Grey matter						
Any anatomical finding	t(13) = −0.39	*p* = 0.70	+0.23	t(13) = 0.91	*p* = 0.38	+1.30
White matter						
Any anatomical finding	t(13) = 0.06	*p* = 0.96	+0.03	t(13) = 0.79	*p* = 0.44	+0.39

a Results of t-statistic are shown.

b Effect sizes are reported with the Cohen d statistic.

Abbreviations: CVR, cerebrovascular reactivity.

In the GM, CVR Z-score decreased linearly with decreasing hematocrit (b = 6.76, *p* = 0.03). This corresponds to a 0.68 decrease in CVR Z-score for each 0.1L/L decrease in hematocrit (Table 4). In the WM, CVR Z-score also decreased linearly with decreasing hematocrit, however it did not reach the boundary of statistical significance (b = 4.23, *p* = 0.30) (Table 5). Similarly, CVR Z-scores decreased linearly with lower hemoglobin, although statistical significance was only demonstrated in grey matter (b = +0.02, *p* = 0.05 and b = 0.01, *p* = 0.40, in GM and WM, respectively). In the WM, none of the biologic and clinical variables examined was significantly associated with the CVR Z-score.

**TABLE 4:** Associations between mean grey matter CVR metrics and clinical and biological variables. Only results of variables that had at least one *p* ≤ 0.2 on univariate analysis in at least one outcome (grey matter CVR or tau) were presented.

	CVR Z-score			Tau Z-score		
	Statistical test(*n*) = statistic^a^	*p*-value	Effect size^b^	Statistical test(*n*) = statistic^a^	*p*-value	Effect size^b^
Age (years)	r(15) = −0.02	0.20	−0.02 per additional year	r(15) = +0.01	0.66	+0.01 per additional year
SCD genotype (SS/Sβ^0^ vs SC/Sβ^+^)	t(13) = −1.41	0.18	+0.76 for SC/Sβ^+^genotype	t(13) = +0.82	0.43	−0.39 for SC/Sβ^+^genotype
Systolic blood pressure (mmHg)	r(15) = +0.01	0.76	+0.07 per 10 mmHg increase in SBP	r(15) = −0.06	0.11	−0.6 per 10 mmHg increase in SBP
Hematocrit (L/L)	r(14) = +6.76	0.03	+0.68 per 0.1 L/L increase in hematocrit	r(14) = 6.73	0.22	−0.67 per 0.1 L/L increase in hematocrit
Hemoglobin (g/L)	r(14) = +0.02	0.05	+0.20 per 10 g/L increase in hemoglobin	r(14) = −0.02	0.23	−0.21 per 10 g/L increase in hemoglobin
White blood cells (10^9^/L)	r(14) = +0.07	0.28	+0.07 per 1 x 10^9^/L increase in WBC	r(14) = −0.19	0.05^c^	−0.19 per 1 x 10^9^/L increase in WBC

a For binary and categorical variables, results of t- and F-statistic are shown, respectively. For continuous variables, unstandardized coefficients are shown.

b For binary and categorical variables, effect sizes are reported with the Cohen d and η2 statistics, respectively.

c Linear correlation for Tau Z-score and neutrophils: r(13) = 0.53, *p* = 0.05; monocytes: r(13) = 0.29, *p* = 0.32; lymphocytes r(13) = 0.35, *p* = 0.21.

Abbreviations: CVR, cerebrovascular reactivity; SBP, systolic blood pressure; SCD, sickle cell disease; WBC = white blood cells.

**TABLE 5:** Associations between mean white matter CVR metrics and clinical and biological variables. Only results of variables that had at least one *p* ≤ 0.2 on univariate analysis in at least one outcome (white matter CVR or tau) were presented.

	CVR Z-score			Tau Z-score		
	Statistical test = statistic^a^	*p*-value	Effect size^b^	Statistical test = statistic^a^	*p*-value	Effect size^b^
Age (years)	r(15) = −0.03	0.18	−0.03 per additional year	r(15) = +0.03	0.37	+0.03 per additional year
SCD genotype (SS/Sβ^0^ vs SC/Sβ^+^)	t(13) = −0.60	0.56	+0.34 for SC/Sβ^+^genotype	t(13) = +0.32	0.75	−0.16 for SC/Sβ^+^ genotype
Systolic blood pressure (mmHg)	r(15) = −0.002	0.95	−0.02 per 10 mmHg increase in SBP	r(15) = −0.06	0.11	−0.43 per 10 mmHg increase in SBP
Hematocrit (L/L)	r(14) = 4.23	0.30	+0.42 per 0.1 L/L increase in hematocrit	r(14) = −3.85	0.47	−0.38 per 0.1 L/L increase in hematocrit
Hemoglobin (g/L)	r(14) = 0.01	0.40	+0.11 per 10 g/L increase in hemoglobin	r(14) = −0.01	0.44	−0.13 per 10 g/L increase in hemoglobin
White blood cells (10^9^/L)	r(14) = 0.11	0.15	+0.11 per 1 x 10^9^/L increase in WBC	r(14) = −0.21	0.02^c^	−0.21 per 1 x 10^9^/L increase in WBC
Hydroxyurea	t(13) = 1.43	0.18	+0.72 for patients on HU	t(13) = +0.52	0.61	−0.29 for patients on HU

a For binary and categorical variables, results of t- and F-statistic are shown, respectively. For continuous variables, unstandardized coefficients are shown.

b For binary and categorical variables, effect sizes are reported with the Cohen d and η2 statistics, respectively.

c Linear correlation for Tau Z-score and neutrophils: r(13) = 0.58, *p* = 0.03; monocytes: r(13) = 0.30, *p* = 0.30; lymphocytes r(13) = 0.50, *p* = 0.07.

Abbreviations: CVR, cerebrovascular reactivity; HU, hydroxyurea; SBP, systolic blood pressure; SCD, sickle cell disease; WBC, white blood cells.

Increasing WBC count was consistently associated with a reduction in tau Z-score in both GM and WM (−0.19 per 1 × 10^9^/L increase in grey matter, *p* = 0.05, -0.21 per 1 × 10^9^/L increase in WM, *p* = 0.02). Unlike CVR however, there was no association between tau and hematocrit or hemoglobin.

#### 3.6.2 Multivariate analysis

In the grey matter, CVR Z-scores were independently associated only with hematocrit, after adjustment for age and SCD genotype (unstandardized beta = 11.75, *p* = 0.1). Hemoglobin was not used for the model, because it was highly collinear with hematocrit. This model accounted for a majority of CVR variability in our sample (*R* ^2^ = 0.66, adjusted *R* ^2^ = 0.48). In the WM, neither age, white blood cells or hydroxyurea were independently associated with CVR in the multivariable model (Table 6).

**TABLE 6:** Associations between grey and white matter CVR and Tau with clinical and biological variables on multivariable analysis. Only associations with *p* ≤ 0.2 were included in the models.

	CVR Z-score			Tau Z-score		
	Unstandardized	*p*-value	VIF	Unstandardized	*p*-value	VIF
Grey matter						
Age	−0.03	0.07	1.01	-	-	-
SCD genotype (SS/Sβ^0^ vs SC/Sβ^+^)	−0.25	0.14	2.4	-	-	-
Systolic blood pressure	-	-	-	−0.05	0.11	1.00
Hematocrit^a^	11.75	0.01	2.4	-	-	-
White blood cells	-	-	-	−0.20	0.03	1.00
Model statistics	R^2^ = 0.60, Adjusted R^2^ = 0.48			R^2^ = 0.43, Adjusted R^2^ = 0.33		
White matter						
Age	−0.01	0.62	1.18	-	-	-
Systolic blood pressure	-	-	-	−0.05	0.07	1.00
White blood cells	0.08	0.25	1.07	−0.22	0.007	1.00
Hydroxyurea	0.66	0.15	1.12		-	-
Model statistics	R^2^ = 0.38, Adjusted R^2^ = 0.20			R^2^ = 0.55, Adjusted R^2^ = 0.47		

a Hemaglobin and hematocrit were highly colinear (VIF = 20–27).

Abbreviations: CVR, cerebrovascular reactivity; SCD, sickle cell disease; VIF, variation inflation factor.

With respect to tau, Z scores in the grey matter were independently associated only with white blood cells, after adjustment for systolic blood pressure (unstandardized beta = −0.20, *p* = 0.03). This model accounted only for part of the tau variability in our sample (*R* ^2^ = 0.43, adjusted *R* ^2^ = 0.33). In the WM, tau Z-scores were also independently associated only with white blood cells, after adjustment for systolic blood pressure (unstandardized beta = −0.22, *p* = 0.007). This model accounted for a majority of the tau variability in our sample (*R* ^2^ = 0.55, adjusted *R* ^2^ = 0.47).

## 4 Discussion

### 4.1 Key results

We measured a reduction in global and regional CVR in both GM and WM, compared to non-SCD controls, in a sample of SCD adults without prior known cerebral vascular abnormalities. This finding highlights the vulnerability of SCD patients to cerebral ischemia, even in the absence of large-vessel cerebral vasculopathy. This data supports the increasing body of evidence demonstrating that SCD patients without overt cerebral vasculopathy are at increased risk of stroke: of the 25 strokes which occurred in a retrospective cohort of about 1,500 SCD children in France, almost half (N = 12) did not have overt cerebral vasculopathy (Linguet et al., 2021). Conversely, cerebral vasculopathy does not completely correlate with increased risk of future strokes or SCI (DeBaun and Kirkham, 2016). Angiographic evidence of large-vessel vasculopathy by computed tomography or magnetic resonance is thus insufficient in predicting future stroke risk in SCD patients and alternative biomarkers of stroke risk prediction is urgently needed. Utilizing the assessment of microvascular function by assessing CVR by standardized hypercapnic isoxic stimulus has the potential to expand our understanding of the pathophysiology of SCI and stroke in these patients. By identifying regional differences in CVR and tau to provide a visual map of areas of cerebral vasculature that are disproportionally at risk of ischemia, we hope to better delineate the pathophysiology of cerebral injury in these patients. The finding of reduced CVR in SCD patients without known large-vessel cerebral vasculopathy indicates that the CBF impediment may be due to microvascular disease as opposed to macrovascular disease.

Risk factors for CVR abnormality in grey and white matters appear to be distinct. The strong association of reduced GM CVR with reduction in hematrocrit, even after adjustment for potential confounders, confirms prior findings (Borzage et al., 19852016; Tanne et al., 2010; Gevers et al., 2012). This could reflect the asymmetric consumption of reserve with a hyperemic response to anemia. Association with genotype would be reflected through its effect on the hematocrit.

The inability to show a statistical association of hematocrit with CVR in WM and tau could be the result of the small range in hematocrit values and the relationship with CVR not being linear over the small range. We must still consider that hematocrit is not the only driver of the adaptation. WM is comprised mostly of small vessels with a small range in flows. Therefore, tau could be a more meaningful metric for WM than CVR. The longer latency in response of CVR may reflect small vessel disease. SCD has multiple risk factors for small vessel disease such as chronic inflammation, genetic fragility, sleep apnea, that were not assessed. Small vascular injury due to repeated vaso-occlusive episodes, episodes of hemodynamic instability (sepsis, acute chest syndrome, multi organ failure) also has also likely accumulated over many years in the patients. The variation in clinical course may also account for variation in imaging findings between patients.

The strong statistical association between white blood cell counts and WM and GM tau was an unexpected finding. This association remained statistically significant even after correcting for potential confounders such as hydroxyurea. Assuming this is not the result of a type 1 error, it would suggest that higher WBC counts, in particular neutrophils, within the normal range, are associated with reduced latency of CVR response. Perhaps the connection is through vascular adhesion, the inflammatory pathway or shared genetic determinants (Reiner et al., 2011). This finding also parallels that by Choi et al., who demonstrated lower white blood cell count in SCD patients was associated with higher mean “deformation index” (DI), a measurement of WM volume shrinkage compared to reference population, in a multivariable regression model (Choi et al., 2019). Similarly, Linguet et al. found stroke in SCD patients without large-vessel vasculopathy was associated with lower WBC count (Linguet et al., 2021).

We illustrate a possible association between the disease-modifying treatment hydroxyurea with CVR in the white matter but not with HbF. Hydroxyurea is known to exert pleiotropic effects on sickle cell disease, in addition to fetal hemoglobin induction. This includes reduced hemolysis, decreased white blood cell and platelet counts, decreased endothelial activation and adhesion, reduced thrombosis and microparticle formation, and reduced vaso-constriction *via* production of nitric oxide by hydroxyurea (Platt, 2008; Green and Barral, 2014)*.*

### 4.2 Rationale in the approach of the hypercapnic stimulus

While other groups have shown reduced and delayed CVR in children using similar measurement techniques (Leung et al., 2016; Kosinski et al., 2017) and in adults using an acetazolamide challenge (Vaclavu et al., 2019), this is the first time where CVR was assessed by standardized hypercapnic isoxic stimulus in SCD patients. Main reasons for choosing this method over acetazolamide or breath-holding are 1) interindividual variability in the magnitude of response, timing and ability to maintain isocapnia making comparison between individuals difficult; 2) tau cannot be assessed using acetazolamide since the time course of change in stimulation and the rise of CO_2_ are considerably longer than those of the vascular responses, substantially obscuring them. On the other hand, the vasodilatory stimulus produced using sequential gas delivery is standardized, controlled, and reproducible. It takes 2 breaths to implement a targeted and stable level of PaCO_2_, unlike acetazolamide. TheThe ability to institute an abrupt onset of the vasoactive stimulus enablesenables the measurement of the speed of the vascular response. ThisThis novel metric of vascular performance is postulated to reflect vessel compliance and functional endothelial integrity. The availability of a standardized repeatable stimulus has the advantages of generating data that repeatable between subjects, and in the same subject over time. The disadvantage is that it requires gas administration or intravenous injection apparatus. The apparatus we use is custom designed for its purpose, and thus is somewhat cumbersome and expensive. The patient CVR and τ results were normalized voxel-wise and expressed as Z scores relative to the mean and standard deviation of the same metric in the corresponding voxels of a previously generated reference atlas of 42 healthy controls. This enables the assessment of particular regions of interest in individual patients with respect to the normality of their measures.

### 4.3 Limitations

First, because of its cross-sectional nature, this study does not allow us to infer causality between patient characteristics and findings on functional brain imaging. Therefore, the biological, clinical and radiologic variables of interest cannot be used to predict the development of reduced and/or future slowed vascular reactivity. Second, the small sample does not provide the necessary power to associate CVR metrics with other patient characteristics. As a result, there is a high risk of type II error and wrongly concluding that there is no association between a variable and CVR metric. However, we had a representative sample with heterogeneous disease characteristics, to allow us to generate hypotheses in our exploratory analysis. Third, we acknowledge the significant risk of multiplicity in our exploratory analysis. We tested for the associations of 19 variables with 2 CVR metrics in 2 anatomical regions (GM and WM). With 76 statistical tests, just by chance, assuming a type I error of 5%, we could generate up 3-4 false positive results. However, the aim of this exploratory analysis was to generate new hypotheses and we did not want to discard possible real associations on the argument of a possible false positive. Fourth, the control atlas did not include anemic individuals of other etiologies. As such, the data is not controlled for hematocrit so causation cannot be inferred. However, Tau and CVR as measures are comparable between groups. This allowed us to study the correlation with hematocrit. Fifth, we selected normal controls that were within the age range the SCD patients (34.3 ± 13.6 years). Regardless of any age differences that may exist, CVR metrics in normal subjects using our protocol are found to be stable across age groups (McKetton et al., 2018). Lastly, we did not quantify volumes of SCI and WMH. While this study introduces vascular function tests on the anticipation that such tests may prove to be predictive of these lesions, this study is not structured or powered to test such predictions.

BOLD MRI was used as a surrogate measure for CBF which requires a number of considerations. First, the BOLD signal increases with CBF as a result of the dilution of the concentration of deoxyhemoglobin with oxyhemoglobin as the oxygen extraction fraction decreases. This in turn produces a non-linear response at very high and low flows (Hoge et al., 1999), which in turn may lead to inaccuracies in CVR measures if assuming a linear model, particularly in the presence of pathology such as steno occlusive disease (Sobczyk et al., 2014). Amplitude and Tau measures were calculated based on the step change in P_ET_CO_2_ starting from each subjects baseline P_ET_CO_2_ and increased by 10 mmHg. Studies have shown that for most individuals the CBF response to PaCO_2_ over the PaCO_2_ range of resting ± 10 mmHg remains fairly in the linear range of the resulting BOLD signal (Bhogal et al., 2016; Duffin et al., 2017). Trancredi and Hoge showed that in the P_ET_CO_2_ range of 40–50 mmHg, CBF as measured by arterial spin labelling remained as a linear function of P_ET_CO_2_ (Tancredi and Hoge, 2013). Second, is the effect of changes in PaCO_2_ on cerebral blood volume (CBV) and the cerebral metabolic rate of oxygen (CMRO_2_). It is assumed that the fractional change in baseline CBV that contributes to the BOLD signal is small compared to that of CBF. It is also assumed that a 10 mmHg increase in CO_2_ does not affect CMRO_2_ (Yezhuvath et al., 2009; Yablonskiy, 2011). Specific artefacts associated with BOLD, such as those arising from differences in baseline metabolism and alterations in CBV (such as increased signal changes over venous pools of blood), contribute to the atlas variability and are therefore controlled by the z-map approach (Sobczyk et al., 2015). A final consideration of the use of BOLD as a surrogate for CBF is the relationship between the changes in BOLD and PaCO2 which is highly dependent on the baseline PaCO_2_ (Cohen et al., 2002; Halani et al., 2015). The strong dependence of CVR on baseline PaCO_2_ as discussed in Sobczyk et al., 2014, was mitigated by setting all subjects to their PaCO_2_ baseline (resting) values and targeting changes in P_ET_CO_2_ relative to their resting.

### 4.4 Future directions

The high risk of ischemic and hemorrhagic stroke with high mortality rate in SCD patients (Ohene-Frempong et al., 1998; DeBaun et al., 2020), the higher prevalence of SCI SCD adults compared to children (Houwing et al., 2020), and higher risk of future infarct recurrence (Jordan et al., 2018; Houwing et al., 2020) and neurocognitive impairment in SCI continues to be major barriers in the treatment of individuals with SCD. Transfusion appears to be ineffective at preventing recurrent stroke in certain patients (Hulbert et al., 2011) while some studies indicate that patients with certain characteristics may not require long-term transfusion (Ware et al., 2016). The SIT trial has shown that 13 SCD children with SCI will need to be treated to prevent one recurrent infarct (SIT trial). Transfusion itself is not without risks, both non-infectious and infectious (Callum et al., 2016) Therefore, blanket deployment of transfusion in SCD patients is neither a cost-effective or safe approach. Large vessel vasculopathy remains a poor predictor of future stroke and SCI risk in SCD patients. At present, the only known risk factor consistent across studies for SCI recurrence and overt stroke in SCD is previous SCI, and majority of these studies were in the pediatric population (Houwing et al., 2020). Thus more precise methods to evaluate and risk stratify this population is needed. CVR using standardized hypercapnic isoxic stimulus may further delineate those at risk of cerebral ischemia and allow a tailored approach in the deployment of disease modifying or curative therapies (e.g., hydroxyurea, transfusion, allogeneic stem cell transplant or gene therapy) in susceptible patients. To achieve this aim however, CVR using this approach will need to be validated in larger cohorts of SCD patients in a longitudinal fashion. The issue of whether simply raising the hematocrit with transfusion will be sufficient to ameliorate the risk of ischemia in SCD patients without large-vessel vasculopathy will also need to be resolved and can be achieved with prospective study of transfusion in this patient population using CVR with this approach. It will also address the question of the relative contributions between anemia and small-vessel vasculopathy from the vaso-occlusive and hemolytic effect of sickle hemoglobin containing red blood cells on the risk of ischemia in SCD patients. For this purpose, a new comparator cohort should be collated of young people with a range of hematocrits due to other genetic (thalassemia), nutrient deficiency (iron, vitamin B12), autoimmune, or bone marrow underproduction etiologies.

## 5 Conclusion

The current study provides evidence of impaired cerebral microvascular function in adult with SCD, relative to population controls, using a standardized and reproducible vasoactive stimulus. Microvascular CVR responses are attenuated in magnitude (too little) and rate of responsiveness (too slow) in both grey and white matter in this cohort of adult SCD patients without known large vessel vasculopathy. The relative contributions between the chronic anemic state versus vaso-occlusion- and hemolysis-driven vasculopathy will be determined in future prospective studies with suitable anemic controls. These findings show that measuring CVR using this approach represents a promising tool to assess disease state. Whether this novel tool can predict stroke risk and therapeutic efficacy disease modifying therapeutics in SCD patients merits further investigation.

## Data Availability

The raw data supporting the conclusion of this article will be made available by the authors, without undue reservation.

## Abbreviations

ACA, anterior cerebral artery; BOLD, blood-oxygenation-level-dependent; CBF, cerebral blood flow; CO_2,_ carbon dioxide; CVR, cerebrovascular reactivity; FLAIR, fluid-attenuated inversion recovery; GFR, globular filtration rate; GM, grey matter; HbF, hemoglobin F; HU, hydroxyurea; IQR, interquartile range; IR-FSPGR, Inversion-Recovery Prepared Fast Spoiled Gradient Echo; LDH, lactate dehydrogenase; MCA, middle cerebral artery; MCV, mean corpuscular volume; MRA, magnetic resonance angiography; MRI, magnetic resonance imaging; PaCO_2_, arterial partial pressure of carbon dioxide; PCA, posterior cerebral artery; P_ET_CO_2_, end-tidal partial pressures of CO_2_; SBP, systolic blood pressure; SCD, sickle cell disease; SCI, silent cerebral infarct; SD, standard deviation; STROBE, Strengthening the Reporting of Observational Studies in Epidemiology; VIF, variation inflation factor; WBC, white blood cells; WM, white matter.
