# Supplementary material for: Sickle cell cerebrovascular reactivity to a CO2 stimulus: Too little, too slow
Source: Front Physiol. 2022 Aug 19;13:886807. doi: 10.3389/fphys.2022.886807 (PMC9437621; doi:10.3389/fphys.2022.886807)
Supplement: Supplementary file 1 [file DataSheet1.docx]

**Supplementary Table 1 :** Resting values for SCD subjects.

|  | **Age Range (years)** | | |
| --- | --- | --- | --- |
| **42 Controls Total** | **18-29** | **30-39** | **40+** |
| **18 females** | 9 | 9 | 0 |
| **24 males** | 9 | 6 | 9 |
| Mean Age (SD): 34.3(13.6)  Abbreviations: SCD = sickle cell disease | | | |
|  | | | |

**Supplementary Table 2 :** Resting P_ET_CO_2_ values for SCD subjects.

|  | Resting P_ET_CO_2_ (mmHg) |
| --- | --- |
| s1 | 35 |
| s2 | 42 |
| s3 | 45 |
| s4 | 41 |
| s5 | 47 |
| s6 | 42 |
| s7 | 37 |
| s8 | 44 |
| s9 | 43 |
| s10 | 41 |
| s11 | 44 |
| s12 | 39 |
| s13 | 36 |
| s14 | 33 |
| s15 | 45 |
| Average (SD) | 40.9+/-4.1 |
| Abbreviations: P_ET_CO_2_ = end-tidal partial pressures of CO_2_, SD = standard deviation. | |

**Supplementary Table 3 :** Regional CVR metrics according to the 3 main vascular territories. Results are shown for grey matter (GM) and white matter (WM).

|  | | ACA | | | | MCA | | | | PCA | | | |
| --- | --- | --- | --- | --- | --- | --- | --- | --- | --- | --- | --- | --- | --- |
|  |  | Left | | Right | | Left | | Right | | Left | | Right | |
|  |  | GM | WM | GM | WM | GM | WM | GM | WM | GM | WM | GM | WM |
| CVR | Mean Z-score (SD) | -0.5 (0.6) | -0.5 (0.6) | -0.5 (0.5) | -0.4 (0.5) | -0.4 (0.6) | -0.5 (0.8) | -0.5 (0.6) | -0.6 (0.7) | -0.7 (0.7) | -0.6 (0.9) | -0.6 (0.9) | -0.6 (0.8) |
|  | [range] | -1.3 to  0.5 | -1.6 to  0.6 | -1.5 to  0.4 | -1.7 to  0.6 | -1.8 to  0.5 | -2.4  to  0.8 | -1.6 to  0.6 | -2.1  to  1.0 | -2.5 to  0.5 | -3.0  to  1.1 | -2.1  to  0.8 | -2.6  to  0.9 |
| Tau | Mean Z-score (SD) | +1.1 (1.1) | +0.9 (0.8) | +1.2 (1.2) | +0.8 (0.9) | +0.9 (1.0) | +0.7 (1.0) | +0.8 (1.0) | +0.5 (1.0) | +0.7 (0.9) | +0.5 (1.1) | +0.7 (1.0) | +0.6 (1.1) |
|  | [range] | -0.4 to 3.8 | -0.4 to 2.7 | -0.4 to 4.0 | -0.6 to 2.9 | -0.6 to 3.4 | -0.7 to 3.0 | -0.5 to 3.3 | -0.8 to 2.6 | -0.5 to 2.9 | -0.7 to 2.6 | -0.5 to 2.9 | -0.7 to 2.6 |
| Abbreviations: ACA = anterior cerebral artery, CVR = cerebrovascular reactivity, GM = grey matter, MCA = middle cerebral artery, PCA = posterior cerebral artery, SD = standard deviation, WM = white matter. | | | | | | | | | | | | | |
